# Incidence, Risk Factors and Consequences of Epilepsy-Related Injuries and Accidents: A Retrospective, Single Center Study

**DOI:** 10.3389/fneur.2018.00414

**Published:** 2018-06-15

**Authors:** Laurent M. Willems, Nina Watermann, Saskia Richter, Lara Kay, Anke M. Hermsen, Susanne Knake, Felix Rosenow, Adam Strzelczyk

**Affiliations:** ^1^Department of Neurology, Epilepsy Center Frankfurt Rhine-Main, Goethe University Frankfurt, Frankfurt, Germany; ^2^Department of Neurology, Epilepsy Center Hessen, Philipps University of Marburg, Marburg, Germany

**Keywords:** epilepsy, seizure, falls, laceration, accident

## Abstract

**Introduction:** This study was designed to evaluate risk factors and incidence of epilepsy-related injuries and accidents (ERIA) at an outpatient clinic of a German epilepsy center providing healthcare to a mixed urban and rural population of over one million inhabitants.

**Methods:** Data acquisition was performed between 10/2013 and 09/2014 using a validated patient questionnaire on socioeconomic status, course of epilepsy, quality of life (QoL), depression, injuries and accidents associated with seizures or inadequate periictal patterns of behavior concerning a period of 3 months. Univariate analysis, multiple testing and regression analysis were performed to identify possible variables associated with ERIA.

**Results:** A total of 292 patients (mean age 40.8 years, range 18–86; 55% female) were enrolled and analyzed. Focal epilepsy was diagnosed in 75% of the patients. The majority was on an antiepileptic drug (AEDs) polytherapy (mean number of AEDs: 1.65). Overall, 41 patients (14.0%) suffered from epilepsy-related injuries and accidents in a 3-month period. Besides lacerations (*n* = 18, 6.2%), abrasions and bruises (*n* = 9, 3.1%), fractures (*n* = 6, 2.2%) and burns (*n* = 3, 1.0%), 17 mild injuries (5.8%) were reported. In 20 (6.8% of the total cohort) cases, urgent medical treatment with hospitalization was necessary. Epilepsy-related injuries and accidents were related to active epilepsy, occurrence of generalized tonic-clonic seizures (GTCS) and drug-refractory course as well as reported ictal falls, ictal loss of consciousness and abnormal peri-ictal behavior in the medical history. In addition, patients with ERIA had significantly higher depression rates and lower QoL.

**Conclusion:** ERIA and their consequences should be given more attention and standardized assessment for ERIA should be performed in every outpatient visit.

## Introduction

Epilepsy is a common and chronic neurological disorder that affects about 39 million people worldwide (1, 2). People with epilepsy are subject to social and vocational stigma, have only restricted access to the labor market and significantly reduced employment opportunities (3). Moreover, quality of life (QoL) is significantly reduced for themselves and their caregivers (4–9). Active epilepsy with persisting seizures is associated with loss of consciousness, uncontrolled movements, falls or periictal abnormal behavior may predispose to accidents and injuries such as burns, contusions, lacerations or fractures (10–12).

A prospective longitudinal analysis from Finland on 245 children with epilepsy since 1964 showed a significantly increased age- and sex-adjusted mortality. During the 40-year follow up, 60 (24%) subjects died and 33 (55%) of these events were attributed to the underlying epilepsy. Besides sudden unexpected death in epilepsy (SUDEP) and status epilepticus, epilepsy-related injuries and accidents (ERIA), such as peri-ictal drowning, were common causes of death. Within this cohort, pneumonia, cardiovascular diseases and suicide have been reported as most frequent causes of death not related to seizures or epilepsy (13–15). ERIA were shown to be a major cost factor for hospitalizations among patients with epilepsy (16). One major problem with ERIA is unreported cases in daily practice. If not investigated in detail, it can be assumed that approximately 50% of ERIA are falsely not documented as seizure-related in medical documentation (17).

The main aim of our study was to assess the frequency and types of ERIA in a cohort of consecutive patients with epilepsy using a validated questionnaire (18) and to search for variables associated with ERIA. Improved screening parameters for ERIA may help in reducing the frequency and severity of ERIA. A second aim was to assess QoL and depression as possible consequences of ERIA.

## Patients and methods

### Study settings, patients and design

This study was performed as a survey at the epilepsy outpatient clinic of the University Hospital Marburg. The University Hospital Marburg is a large multispecialty tertiary care hospital in the center of Germany that provides healthcare to a population of over 1,000,000 inhabitants. Marburg is located within the postal code area 35, which was used previously for a population-based estimation of the incidence of status epilepticus and different cost-of-illness studies (3, 19–21). After receiving written informed consent, all adult epilepsy patients aged 18 years or older were eligible. Socioeconomic data and information on injuries or accidents as well as on QoL were accessed between 10/2013 and 09/2014 using a validated questionnaire (18). Patients were asked to complete the questionnaire concerning their individual epilepsy related experiences during the last 3 months. The diagnosis was based on the definitions proposed by the International League Against Epilepsy and the International Bureau for Epilepsy (22) and was provided by the treating physician. Any patient with at least one seizure during the last 12 months at enrolment was classified as having an “active epilepsy.” Patients were excluded when the diagnosis of epilepsy could not be determined without doubt. The study had the approval of the local ethics committee.

### Variables associated with epilepsy-related injuries and accidents

Specification of epilepsy syndromes as well as information regarding intake of AEDs was assessed by the attending physician. The following factors were identified as possible parameters influencing injuries or accidents in patients with epilepsy according to published literature (18): patient age, duration of epilepsy, sex, epilepsy syndrome, postictal patterns of behavior as well as seizure frequency and semiology (loss of consciousness, nocturnal seizures). Abnormal postictal behavior patterns were classified in line with the guidelines of the German employers' liability insurance association (Unfallversicherung, DGUV 250-001), which classifies seizures with abnormal postictal behavior as “high risk” for related injuries or accidents at work (23). Refractory epilepsy was classified according to ILAE definitions (24). Moreover, different sociodemographic and clinical characteristics were documented.

### Changes in mood and quality of life due to ERIA

To evaluate the psychological aspects of ERIA, various established neuropsychological inventories were assessed using already established questionnaires: To measure health related QoL, the QOLIE31 score (Quality of Life in Epilepsy Inventory) (25) and EQ5D score (Euro Quality of Life) were used (26). For EQ5D, both relevant parameters, i.e., overall TTO (time trade-off) and VAS (visual analog scale), were determined (26). To evaluate health-related depression, NDDI-E (Neurological Disorders Depression Inventory for Epilepsy) (27) and BDI-II (Becks Depression Inventory) were used (28). As an independent parameter, ABNAS (A-B Neuropsychological Assessment Schedule) was assessed (29). Liverpool Adverse Events Profile (LAEP) was measured to detect AED-specific aspects in QoL (30, 31).

### Data entry and statistical analysis

Data entry and analysis were performed using the File Maker Pro 8.5 database (Filemaker Inc., Santa Clara, CA, USA). A double-entry procedure was employed to assure a high level of data accuracy. Statistical analyses were performed using IBM SPSS Statistics 22 (IBM Corporation, Armonk, NY, USA). For the calculation of variables that potentially influence injuries and accidents in patients with active epilepsy, a chi-square test following Pearson and Benjamini-Hochberg-Adjustment was used to exclude multiple testing bias (32, 33). In addition, regression analysis was performed. *P*-values < 0.05 were considered as significant.

## Results

### Sociodemographic and clinical characteristics

We enrolled 292 patients with epilepsy and a mean age of 40.8 years (SD ± 15.6; range 18–86 years). Gender ratio was nearly balanced with 55% female patients. Two-thirds of patients (67.8%) had an active epilepsy with at least one seizure within the last 12 months. The majority of 75% (*n* = 219) suffered from focal epilepsy (FE), 19.5% (*n* = 57) from an idiopathic generalized epilepsy (IGE) and, in 5.5%, the epilepsy syndrome was reported as unclassified; details are presented in Table 1. Between the two cohorts with IGE or FE, there were no significant differences in gender distribution, number of AED or seizure freedom. The mean age in the IGE cohort was significantly younger. Detailed information of sociodemographic and clinical characteristics for all patients and the FE and IGE cohorts are displayed in Table 2.

**Table 1:** Epilepsy syndromes within the cohort.

**Epilepsy syndrome (*n* = 292)**	**% (*n*)**
Focal epilepsy	75.0(219)
TLE	49.0(143)
FLE	10.6(31)
Other lobes	5.1(15)
Multilobar	4.5(13)
Other/cryptogenic	5.8(17)
Idiopathic generalized epilepsy	19.5(57)
IGE with grand mal on awakening	0.3(1)
JME (Janz syndrome)	4.8(14)
Juvenile absence epilepsy	1.7(5)
Other	12.7(37)
Unclassified	5.5(16)

*TLE, temporal lobe epilepsy; FLE, frontal lobe epilepsy; IGE, idiopathic generalized epilepsy; JME, juvenile myoclonic epilepsy*.

**Table 2:** Sociodemographic and clinical characteristics of the entire cohort and for patients with focal and idiopathic generalized epilepsies.

		**Total *n* = 292**	**Focal epilepsy *n* = 219**	**IGE *n* = 57**	***p*-value**
Age in years^a^		40.8 ± 15.6	43.1 ± 15.5	31.4 ± 12.5	<0.001^b^
		Range:18–86	Range: 18–86	Range: 18–63	
Disease duration in years^a^		14.2 ± 19.8	14.3 ± 14.1	14.7 ± 13.6	0.881^b^
		Range: 0.1–63	Range: 0.1–63	Range: 0.1–55	
Sex					
Male		45.2 (132)	46.1 (101)	40.4 (23)	0.121^c^^,^^d^
Female		54.9 (160)	53.9 (118)	59.7 (34)	
Anticonvulsants	Mean no of AED	1.6 (±0.8)^a^	1.7(±0.8)^a^	1.5 (±0.6)^a^	
		*% (n)*	*% (n)*	*% (n)*	
	No AEDs	1.7 (5)	0.18 (4)	0.3 (1)	0.958^c^
	Monotherapy	48.3 (141)	45.7 (100)	54.4 (31)	
	2 AEDs	34.6 (101)	34.7 (76)	38.6 (22)	
	≥3 AEDs	15.4 (45)	17.8 (39)	7.0 (4)	
Seizures		% (*n*)	% (*n*)	% (*n*)	
Seizure freedom >1 year		32.2 (94)	29.2 (64)	45.6 (26)	0.019^c^
Active epilepsy		67.8 (198)	70.8 (155)	54.5 (31)	

a *Mean ± standard deviation*.

b *p-value calculated using T-Test*.

c *p-value calculated using chi2-test*.

d *Male vs. female*.

The five most frequently prescribed AEDs were levetiracetam (LEV, 53.1%), lamotrigine (LTG, 35.6%) and valproate (VPA, 16.1%) followed by carbamazepine (CBZ) and lacosamide (LCM) (each 11.6%). A wide variability of newer AEDs (second and third generation) were used, while 28.8% of prescribed AEDs were so called “old” substances like VPA, CBZ, phenobarbital (PB) and phenytoine (PHT). Only 13.7% of the AEDs had enzyme-inducing properties (i.e., CBZ, PB, primidone, PHT). For a more detailed listing, please refer to Table 3.

**Table 3:** Prescription pattern of anticonvulsant drugs within the cohort.

**AED**	**% Total patients (*n*)**
Levetiracetam	53.1(155)
Lamotrigine	35.6(104)
Valproate	16.1(47)
Carbamazepine	11.6(34)
Lacosamide	11.6(34)
Zonisamide	9.2(27)
Oxcarbazepine	7.5(22)
Perampanel	6.2(18)
Topiramate	3.8(11)
Pregabalin	2.4(7)
Gabapentin	1.7(5)
Clonazepam	1.0(3)
Primidone	1.0(3)
Ethosuximide	1.0(3)
Phenobarbital	0.7(2)
Eslicarbazepine	0.3(1)
Stiripentol	0.3(1)
Methsuximide	0.3(1)
Phenytoine	0.3(1)
Piracetam	0.3(1)
Percentage of “old” AEDs^a^	28.8%
Percentage of enzyme-inducing AEDs^a^	13.7%

a *Enzyme-inducing AEDs: carbamazepine, phenobarbital, primidone, and phenytoin; “old” AEDs: valproate, carbamazepine, phenobarbital and phenytoine*.

### Clinical characteristics of patients with epilepsy-related injuries and accidents

In total, 41 patients (14.0%) suffered from epilepsy-related injuries and accidents (ERIA) within the 3-month observation period. Of these, 20 patients (6.8% of total cohort) required hospitalization. Out of a total of 292 patients, two independent injuries were reported in two cases and one patient suffered from three independent injuries. Besides lacerations (*n* = 18, 6.2%), abrasions and bruises (*n* = 9, 3.2%), fractures (*n* = 6, 2.1%) and burns (*n* = 3, 1.0%), 17 other mild injuries (5.8%) were reported. Among the patients with injuries, 48.8% were male, the mean age was 39.2 years (SD ± 14.1, range 18–67 years). Mean disease duration in patients with ERIA was 14.7 years (SD ± 15.2, range 0.1–40 years). Only one patient in this group reported no AED therapy, 14 participants (14/41; 34.2%) were under anticonvulsant monotherapy, 26 reported taking two or more AEDs (63.4%), and the mean number of AEDs was 2.0 (SD 1.0, range 0–4). The majority of seizure-related injuries was observed in patients suffering from generalized tonic-clonic seizures (*n* = 24, 58.5%, i.e., bilateral tonic-clonic seizures). Another 14 patients (34.2%) reported predominantly simple partial seizures (SPS, i.e., focal seizures with preserved awareness) or complex partial seizures (CPS, i.e., focal onset seizures with impaired awareness); in three cases (7.3%), the habitual seizure type was not stated. In nearly all cases (40 of 41 patients, 97.6%) suffering from ERIA, certain semiological features were reported. Most of the injured patients reported ictal loss of consciousness (*n* = 39, 95.1%) or ictal falls (*n* = 36, 87.8%) or both (*n* = 35, 85.4%). Isolated peri-ictal abnormal behavior (*n* = 14, 34.2%) was less often seen in patients with ERIA, but several patients reported periictal abnormal behavior in association with ictal falls and ictal loss of consciousness (*n* = 12, 29.3%). Only two patients with ERIA reported to suffer from sleep-bound seizures only (4.9%). For a detailed list of all patients that reported ERIA see Table 4.

**Table 4:** Overview of patients with epilepsy-related injuries and accidents (ERIA, *n* = 41).

**Epilepsy**			**Patient**	**Semiology**				**Potential associated factors**				**Injuries and accidents**					
**Duration (y)**	**FE/IGE**	**AEDs (n)**	**Decade of life**	**SPS**	**CPS**	**sGTCS**	**pGTCS**	**Ictal falls**	**Ictal loss of consciousness**	**Abnormal postictal behavior**	**Sleep-associated seizures**	**Fracture**	**Burn**	**Laceration**	**Sprain/bruise**	**Mild injuries**	**Hospitalization required**
–	FE	3	61–70					x	x			x		x			x
0.1	IGE	1	11–20				x	x	x					x			
0.1	FE	1	31–40	x		x		x	x							x	x
0.1	IGE	1	21–30				x	x	x							x	
1	FE	1	51–60					x	x						x		x
1	FE	1	51–60			x		x	x			x					x
1	FE	2	51–60			x		x	x						x		
1	FE	3	21–30		x	x		x	x						x		
1	FE	1	11–20	x		x		x	x						x		
2	FE	3	61–70		x			x	x			x	x				x
2	FE	2	31–40	x				x	x	x					x	x	
4	FE	1	11–20	x		x		x	x							x	x
5	FE	1	61–70	x		x		x	x							x	x
5	IGE	1	11–20		x				x					x			
6	FE	2	31–40		x			x	x	x				x			
7	FE	2	31–40			x		x	x	x				x			
7	IGE	2	41–50	x				x	x							x	
7	FE	1	11–20			x	x		x		x				x		
7	FE	3	41–50			x		x	x	x				x			
8	FE	2	21–30	x				x	x			x					
10	IGE	3	21–30			x			x	x			x	x		x	x
11	FE	3	21–30	x				x	x	x		x		x			x
11	FE	1	21–30	x		x										x	
11	FE	3	31–40	x				x	x	x						x	x
11	FE	3	21–30	x		x		x							x		
13	FE	2	51–60	x				x	x	x				x		x	
13	FE	3	31–40	x				x	x	x				x			x
13	FE	0	41–40	x		x		x	x					x			x
18	FE	3	31–40	x				x	x	x						x	
18	IGE	1	21–30	x		x		x	x					x			
22	FE	3	41–50				x	x	x					x		x	
26	FE	1	31–40			x			x	x	x					x	x
27	FE	3	41–50			x		x	x	x						x	
28	FE	3	41–50	x				x	x	x						x	
29	IGE	2	31–40	x		x		x	x			x		x			x
29	FE	2	41–50			x		x	x					x	x		
37	FE	3	41–50	x		x		x	x							x	x
41	FE	3	51–60	x				x	x	x			x	x			x
48	IGE	4	41–50	x		x		x	x					x		x	
53	FE	1	51–60					x	x					x			
55	FE	3	61–70		x			x	x						x		x

*AEDs, anticonvulsant drugs; SPS, simple partial seizures; CPS, complex partial seizures; sGTCS, secondary generalized tonic-clonic seizures; pGTCS; primary generalized tonic-clonic seizures; mild injuries: biting of lips and tongue, small scrapes, mild contusion, myogelosis, aching muscles*.

### Variables associated with epilepsy-related injuries and accidents

Reported sociodemographic and clinical characteristics were analyzed as potential variables associated with ERIA. Comparing patients with (*n* = 41, 14.0%) to patients without (*n* = 251, 86.0%) ERIA, we identified an active epilepsy (*p* < 0.001), regular GTCS (*p* < 0.001) and a refractory course of disease (*p* = 0.005) as variables associated with ERIA using a univariate analysis. Moreover, ictal falls (*p* = 0.012), ictal loss of consciousness (*p* = 0.022) and inadequate postictal behavior (*p* = 0.017) were significantly increased in patients suffering ERIA. After *post hoc* multivariate analysis all reported parameters remained significant. No difference was determined for patient age, duration of epilepsy, gender, epilepsy syndrome or sleep-related seizures in our cohort; for details please refer to Table 5. Multiple regression analysis revealed a strong positive correlation of ERIA with active epilepsy (*p* = 0.006, *B* = 0.140), all other tested characteristics (i.e., age, sex, disease duration, seizure semiology, epilepsy syndrome, refractive epilepsy) did not reach levels of significance.

**Table 5:** Frequency and clinical characteristics of patients with epilepsy-related injuries and accidents (*n*_total_ = 292, *n*_ERIA_ = 41).

		**Epilepsy related injuries and accidents**					
		**No *n* (%)**	**Yes *n* (%)**	**Total**	**Chi-square**	***p*-value**	***p*-value corrected**
Age	≥50years	82(87.2)	12(12.8)	94	0.2310	0.631	0.688^c^
	<50 years	166(85.1)	29(14.9)	195			
Duration of epilepsy	≥10 years	114(84.4)	21(15.5)	135	0.4000	0.527	0.688^c^
	<10 years	128(87.1)	19(12.9)	147			
Sex	Male	110(84.6)	20(15.4)	130	0.2784	0.598	0.688^c^
	Female	138(86.8)	21(13.2)	159			
**EPILEPSY SYNDROME**							
Focal epilepsy		184(84.8)	33(15.2)	217	0.8103	0.368	0.688^c^
IGE		51(89.5)	6(10.5)	57			
**NEWLY DIAGNOSED EPILEPSY**^a^							
	Yes	16(84.2)	3(15.8)	19	0.0429	0.836	0.688^c^
	No	232(85.9)	38(14.1)	270			
**SEIZURE FREQUENCY**							
Seizure free > 1 year	Yes	91(96.8)	3(3.2)	94	13.8347	<0.001	0.006^c^
	No	157(80.5)	38(19.5)	195			
No GTCS > 1 year	Yes	179(92.3)	15(7.7)	194	20.1980	<0.001	0.006^c^
	No	69(72.6)	26(27.4)	95			
Refractory epilepsy^b^	Yes	57(76.0)	18(24.0)	75	8.0117	0.005	0.02^c^
	No	191(89.3)	23(10.7)	214			
**SEIZURE SEMIOLOGY**							
Ictal falls	Yes	169(82.4)	36(17.6)	205	6.2858	0.012	0.036^c^
	No	77(93.9)	5(6.1)	82			
Ictal loss of consciousness	Yes	198(83.5)	39(16.5)	237	5.2314	0.022	0.044^c^
	No	48(96.0)	2(4.0)	50			
Inadequate peri-ictal behavior	Yes	44(75.9)	14(24.1)	58	4.3352	0.017	0.036^c^
	No	202(88.2)	27(11.8)	229			
Nocturnal seizures only	Yes	6(75.0)	2(25.0)	8	0.8299	0.362	0.688^c^
	No	240(86.3)	38(13.7)	278			

a *Newly diagnosed epilepsy, new onset epilepsy with ≥ 2 seizures within the last 12 months*.

b *According to ILAE guidelines, IGE, idiopathic generalized epilepsy; GTCS, generalized tonic clonic seizure*.

c *Adjusted for multiple testing using Benjamini-Hochberg-Adjustment*.

### Epilepsy-related injuries and accidents are accompanied by a decreased quality of life (QoL)

People suffering from ERIA reported significantly more symptoms indicative of a manifest depression (BDI ≥ 14 [*p* = 0.021], NDDIE ≥ 13 [*p* = 0.002]). Patients with ERIA (overall T 38.9 ± 9.0) had a significant lower (QoL) compared to patients without ERIA (overall T 49.2 ± 10.4 [*p* < 0.001]) in QOLIE-31. Regarding each single item of the QOLIE-31 inventory, patients with ERIA showed a significantly increased seizure worry (*p* ≤ 0.001) and decreased overall QoL (*p* < 0.001), emotional well-being (*p* = 0.001) and energy level (*p* = 0.019) as well as cognitive (*p* < 0.001) and social functions (*p* < 0.001). Medication effects did not reach any level of significance in QOLIE-31 (*p* = 0.68), even if the results for Liverpool Adverse Events Profile (LEAP) were significantly different between patients with and without ERIA (*p* = 0.006). Similar results for QoL were obtained in EQ5D Score [VAS (visual analog scale) 56.0 ±19.1 vs. 68.4 ±18.7, *p* < 0.001]. In addition, ABNAS showed a significant distinction between patients with (22.2 ± 15.7) and without ERIA (35.8 ± 17.9, *p* < 0.001). After *post hoc* multivariate analysis all reported parameters remained significant. For a detailed statistical analysis of QoL and depression scores please refer to Table 6.

**Table 6:** Changes in mood and quality of life due to epilepsy-related injuries and accidents (*n*_total_ = 292, *n*_*ERIA*_ = 41).

		**Epilepsy related injuries and accidents**					
		**No**	**Yes**	**Total**	**Chi-square**	***p*-value**	***p*-value corrected**
**DEPRESSION**							
BDI	Depression	41 (75.9)	13 (24.1)	54	5.3325	0.021	0.021^a^
	(Score ≥ 14)						
	No depression	207 (88.1)	28 (11.9)	235			
	(Score < 14)						
NDDI-E	Major depression	34 (72.3)	13 (27.7)	47	9.7522	0.002	0.003^a^
	(Score ≥ 13)						
	No major depression	154 (90.1)	17 (9.9)	171			
	(Score < 13)						
		**All patients**	**Patients with ERIA**	**Patients without ERIA**	***p*****-value**		
**QUALITY OF LIFE**							
QOLIE-31	Overall T (mean ±SD)	48.1 ± 10.8	38.9 ± 9.0	49.2 ± 10.4	<0.001	0.002^a^	
	Range	17–68	22–56	17–68			
EQ5D	VAS (mean ± SD)	66.7 ± 19.3	56.0 ±19.1	68.4 ± 18.7	<0.001	0.002^a^	
	Range	10–100	10–90	20–100			
**MEDICATION**							
LEAP	Total (mean ± SD)	39.9 ± 10.8	39.2± 10.9	44.6 ± 9.3	0.007	0.008^a^	
	Range	19–64	19–64	26–63			
**OTHERS**							
ABNAS	Total (mean ± SD)	24.0 ± 16.7	35.8 ±17.9	22.2 ± 15.7	<0.001	0.002^a^	
	Range	range 0–72	range 1–72	range 0–63			

*NDDI-E, Neurological Disorders Depression Inventory for Epilepsy; BDI, Becks depression inventory; QOLIE31, Quality of Life in Epilepsy Inventory; ABNAS, A-B Neuropsychological Assessment Schedule; EQ5D, Euro Quality of Life; LEAP, Liverpool Adverse Events Profile; a adjusted for multiple testing using Benjamini-Hochberg-Adjustment*.

## Discussion

This study analyzed in detail a cohort of epilepsy patients in Germany showing the high burden imposed on patients due to ERIA. In addition, patients with ERIA showed a decreased QoL and increased depression rates. Our results are in line with other studies on ERIA, showing that number and frequency of injuries and accidents are significantly increased in epilepsy patients compared to matched controls. However, there are certain limitations of the study design (e.g., a missing epidemiologic approach) that may display limitations of the acquired data and findings. The finding that most enrolled patients had focal and active epilepsy may also affect the comparability with other studies.

In a large database study published in 2010, 8,890 subjects with epilepsy were analyzed, resulting in a 1-year incidence of one or more injuries in 20.6% of patients with and 16.1% of patients without epilepsy (*p* < 0.001) (34). Active epilepsy and refractory course of disease, as demonstrated in our cohort, are variables associated with ERIA. Within the 3-month observation period, 41 of 292 patients (14.0%) reported ERIA. The most common symptoms were lacerations (*n* = 18, 6.2%) followed by abrasions and bruises (*n* = 9, 3.1%), fractures (*n* = 6, 2.1%) and burns (*n* = 3, 1.0%). In 20 cases, urgent medical treatment and hospitalization was necessary. These results are well in line with previously published data on ERIA from a pilot study from 2008 among our outpatients (18, 35) and other international publications reporting ERIA in children, adolescents and adults with epilepsy in Nigeria, the United States, Australia and the UK (34, 36–41). In addition, a study reporting on 52 patients with refractory focal epilepsy led to comparable results with epilepsy-related injuries in 21% of cases with TLE and 8% with other focal epilepsies within a mean disease duration of 22 and 17 years. Lifetime prevalence of ERIA was estimated to 57%. Moreover, this study reported that only 45% of ERIA in general, and especially severe ones, had been documented (17). This is in contrast to another study with 247 enrolled patients showing a rather low frequency for ERIA of 1 per 44 patient years and which claimed a minor severity for the most reported cases (41).

Another prospective study analyzed ERIA using a control group of healthy relatives, showing that epilepsy patients have a 21% higher likelihood to harm themselves over a 1–2 year observation period, however, mainly mild and trivial injuries were reported (42). In children aged 2–16 years, ERIA were reported in 4.7% (43) and children with epilepsy were shown to have a 18–23% increased risk of fracture, a 49% increased risk of thermal injury and more than twice the risk of poisoning from medicinal products (39). Differences between the mentioned studies are probably due to diverging study designs (prospective vs. retrospective), different cohort characteristics and may be influenced by local factors as well as awareness and ascertainment issues. ERIA are commonly underreported and there is a high estimated number of underreported cases (17). The high frequency of ERIA in our study can be attributed to the targeted questions and precise documentation reducing underascertainment.

The vast majority of patients reporting ERIA (96.0%) presented specific ictal features and we were able to identify a significant correlation between ERIA with epilepsy characteristics and semiological aspects. Variables associated with ERIA were active epilepsy, drug-refractory course as well as reported ictal falls and ictal loss of consciousness or abnormal peri-ictal behavior in the medical history. These findings are similar to the results of past studies (18). Moreover, the presence of GTCS was significantly associated with ERIA, which is in line with previous studies, in which over 80% of ERIA occurred during GTCS (41). Additionally, we were able to show a significant coherence between ERIA and abnormal peri-ictal behavior, which was previously controversially discussed (18). Patients with frequent and prolonged ictal auras seem to be at lower risk, which is probably based on the possibility of informing bystanders, calling for help and creating a secure surrounding. As another high-risk subgroup, individuals with active epilepsy could be identified. There was no significant difference in the occurrence of ERIA between patients with FE or IGE, which is surprising because of the common presence of GTCS in patients with IGE. Overall, 75% of IGE patients presented with GTCS compared to 51% with FE in an ERIA cohort (40).

A second focus of our study was to assess QoL and depression as possible consequence of ERIA using a battery of neuropsychological inventories (BDI-II, NDDIE, EQ5D, QOLIE-31, ABNAS, and LEAP) that have been already established and validated in patients with epilepsy (25–29). Subjects reporting ERIA showed significantly increased depression rates and had a lower QoL, which has not been reported so far. The association of lower QoL and a trend to depression with drug-refractory epilepsy has been already shown (12, 27, 28, 31, 44, 45) and ERIA might be associated or rather aggravate these aspects. However, this study was not designed to further analyse and correlate these aspects. Also, adverse effects due to AEDs were significantly higher in patients with ERIA.

The most practical solution to injury prevention is a better seizure control (40, 41). However, this may not be achieved, especially in patients with drug-refractory epilepsies who have been shown to be a high-risk group for ERIA. Therefore, another focus should aim at reducing the risk for ERIA. We propose a prophylactic pathway focusing on three major aspects: (1) identifying high risk patients by a standardized assessment of ERIA, (2) optimising antiepileptic therapy, and (3) developing individual safety assessments to reduce the future risk of ERIA (see Table 7). Individual safety assessments may contain information and education on the risks and consequences of ERIA, possibilities to avoid injuries (behavior, early detection) and suggestions for auxiliary means (helmets, hip protectors, in-house emergency calls, wearables). Even if the majority of epilepsy-related injuries and accidents occur at home (88%) and not in a public space (10%), a professional and epilepsy-specific assessment of the actual or aspired profession and the concomitant work environment seems to be unavoidable, hence, the fact that only 2% of epilepsy-related injuries and accidents occur at work (46, 47).

**Table 7:** Standardized assessment and management of variables associated with ERIA.

**(1) Structured assessment of ERIA**	**(2) Individual evaluation of variables associated with ERIA**	**(3) Development of individual avoidance strategies for ERIA**
**Medical history**	**Seizure semiology**	**Information and consultation**
Short-term: “*Have you had any injury or accident since the last visit?”* “*Was this injury related to an epileptic seizure”*	Generalized tonic clonic seizures^b^ Status epilepticus^a^ Peri-ictal falls^b^ Peri-ictal loss of consciousness^b^ Peri-ictal inadequate behavior^b^	Average risk for ERIA Individual risk for ERIA due to Medical history Seizure semiology Epilepsy syndrome Possible consequences
Long-term: “*Have you ever had an injury or accident that was related to an epileptic seizure?”*	**Epilepsy syndrome**	**Avoiding strategies**
	Active epilepsy^b^ Refractory epilepsy^b^	Safe surrounding Information of caretakers Early detection of seizures
**Examination**		**Auxiliary means**
“*Where have you got this scar/wound from?”* “*Was the injury related to an epileptic seizure?”*		Protective helmets Hip protectors In-house-emergency calls Wearables Seizure dog

*ERIA epilepsy-related injuries and accidents*.

a *Variables associated with ERIA proposed by other publications*.

b *Variables associated with ERIA according to our cohort*.

## Conclusion

ERIA, as well as their clinical consequences, are an underreported but highly relevant aspect in the treatment of patients with active epilepsy. Moreover, ERIA might be associated or rather aggravate neuropsychological symptoms such as depression or decreased QOL in this severely affected subgroup of patients with epilepsy. However, ERIA are only sporadically considered in the development of tailored anti-epileptic therapies. More effort should be spent on the identification and assessment of ERIA or patients at risk as well as on prevention and general education on this topic to increase patient safety, satisfaction and QoL.

## Author contributions

LW and AS generated the research idea, study design, and concept. NW, SR, and AS acquired the data. LW and AS analyzed the data and drafted the work. LW, NW, SR, LK, AH, SK, FR, and AS made critical revisions for important intellectual content and interpreted the data. LW and AS wrote the manuscript. LW, NW, SR, LK, AH, SK, FR, and AS approved the final manuscript.

### Conflict of interest statement

SK reports personal fees from Desitin, Eisai, UCB, as well as support for scientific meetings from AD-tech, Desitin, Eisai, GW pharma, LivaNova, Nihon Kohden and Novartis outside the submitted work. FR reports personal fees from Eisai, grants and personal fees from UCB, grants and personal fees from Desitin Arzneimittel, personal fees and other from Novartis, personal fees from Medronic, personal fees from Cerbomed, personal fees from ViroPharma and Shire, grants from European Union, grants from Deutsche Forschungsgemeinschaft, outside the submitted work. AS reports personal fees and grants from Desitin Arzneimittel, Eisai, LivaNova, Sage Therapeutics, UCB Pharma and Zogenix, outside the submitted work. The remaining authors declare that the research was conducted in the absence of any commercial or financial relationships that could be construed as a potential conflict of interest.
